# Machine Learning-Accelerated First-Principles Study of Atomic Configuration and Ionic Diffusion in Li_10_GeP_2_S_12_ Solid Electrolyte

**DOI:** 10.3390/ma17081810

**Published:** 2024-04-15

**Authors:** Changlin Qi, Yuwei Zhou, Xiaoze Yuan, Qing Peng, Yong Yang, Yongwang Li, Xiaodong Wen

**Affiliations:** 1 State Key Laboratory of Coal Conversion, Institute of Coal Chemistry, Chinese Academy of Sciences, Taiyuan 030001, Chinawxd@sxicc.ac.cn (X.W.); 2 University of Chinese Academy of Sciences, No. 19A Yuquan Road, Beijing 100049, China; 3 National Energy Center for Coal to Clean Fuels, Synfuels China Co., Ltd., Huairou District, Beijing 101400, China; 4 State Key Laboratory of Nonlinear Mechanics, Institute of Mechanics, Chinese Academy of Sciences, Beijing 100190, China; 5 Center of Materials Science and Optoelectronics Engineering, University of Chinese Academy of Sciences, Beijing 100049, China; 6 Guangdong Aerospace Research Academy, Guangzhou 511458, China

**Keywords:** Li_10_GeP_2_S_12_ solid electrolyte, first-principles calculation, Ewald-summation-based electrostatic energy, machine learning- and active-learning-based LAsou method, ab initio molecular dynamics

## Abstract

The solid electrolyte Li_10_GeP_2_S_12_ (LGPS) plays a crucial role in the development of all-solid-state batteries and has been widely studied both experimentally and theoretically. The properties of solid electrolytes, such as thermodynamic stability, conductivity, band gap, and more, are closely related to their ground-state structures. However, the presence of site-disordered co-occupancy of Ge/P and defective fractional occupancy of lithium ions results in an exceptionally large number of possible atomic configurations (structures). Currently, the electrostatic energy criterion is widely used to screen favorable candidates and reduce computational costs in first-principles calculations. In this study, we employ the machine learning- and active-learning-based LAsou method, in combination with first-principles calculations, to efficiently predict the most stable configuration of LGPS as reported in the literature. Then, we investigate the diffusion properties of Li ions within the temperature range of 500–900 K using ab initio molecular dynamics. The results demonstrate that the atomic configurations with different skeletons and Li ion distributions significantly affect the Li ions’ diffusion. Moreover, the results also suggest that the LAsou method is valuable for refining experimental crystal structures, accelerating theoretical calculations, and facilitating the design of new solid electrolyte materials in the future.

## 1. Introduction

In recent years, there has been a growing demand for large-scale rechargeable batteries in power networks and electric vehicles [1,2]. However, safety incidents involving the high-capacity lithium-ion batteries used in these applications have raised concerns due to the use of flammable liquid organic electrolyte solvents. Therefore, researchers have been focusing on developing all-solid-state batteries (ASSBs) as a safer and non-flammable alternative technology [3]. The development of solid-state electrolytes (SSEs) poses a significant challenge in the development of ASSBs. SSEs provide several advantages, such as preventing side reactions, leakage, corrosion, and other undesirable effects associated with liquid electrolytes. Due to their enhanced safety and compactness, solid electrolytes are widely regarded as the preferred materials for constructing the next generation of lithium batteries [4].

**Figure 1:**
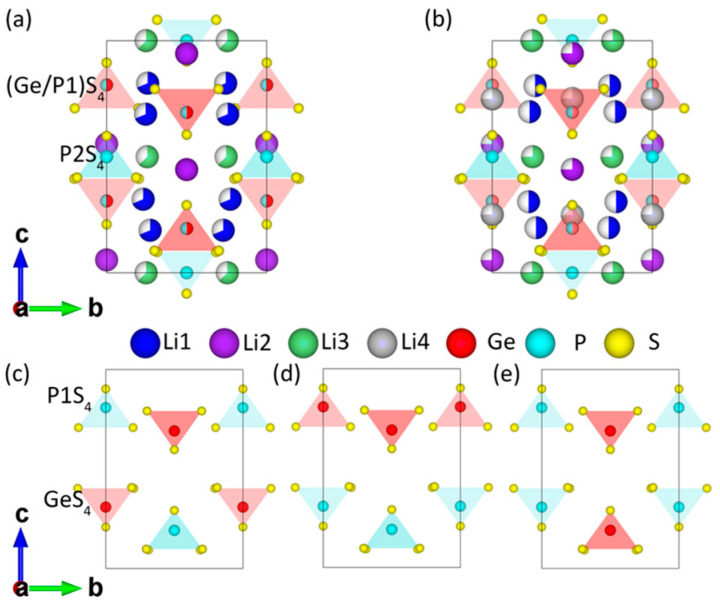
The experimental crystal structures of LGPS (**a**) reported by Kamaya et al. without Li4 sites and (**b**) reported by Kuhn et al. with Li4 sites, and three types of skeletons for (**c**) Zig with a zigzag in the unit cell, (**d**) Pa parallel to the a-axis, and (**e**) Pc parallel to the c-axis.

In 2011, Kamaya et al. reported a novel solid-state electrolyte, Li_10_GeP_2_S_12_ (LGPS), produced in an experiment [5]. At room temperature, LGPS exhibits a high conductivity of up to 12 mS/cm, which is comparable to that of liquid electrolytes. Its crystal structure is shown in Figure 1a. Within the unit cell, there are three types of Li ions, among which Li1 and Li3 occupy fractional positions in the c-channel and Li2 fully occupies the ab-plane. Two types of P ions exist as well, among which P1 co-occupies with Ge to form a (Ge/P1)S_4_ tetrahedron, and the fully occupied P2 and S form the P_2_S_4_ tetrahedron. In 2012, Adams and Rao predicted the existence of new Li4 sites through atomic-scale molecular dynamics (MD) simulations [6]. This prediction was later experimentally confirmed by Kuhn et al. in 2013 through single-crystal XRD characterization, as shown in Figure 1b [7]. Detailed information on the crystal structures reported by Kamaya et al. and Kuhn et al. can be found in Table S1. As characterization techniques continue to improve, richer and more profound information on the distribution of Li ions has been obtained [8,9,10,11]. According to Kato et al., obtaining an LGPS structure with high accuracy can be achieved by taking measurements of a large single crystal with a high-throughput neutron source [12]. Due to the relatively low atomic mass of Li, determining the exact positions and fractional occupancies of Li ions sites is extremely challenging, which limits our understanding of the distribution and local structure of Li ions and further hinders our comprehension of the diffusion mechanisms in LGPS [11].

As for theoretical studies, many attempts have been made to address fundamental issues about the distribution and atomic configuration (or arrangement) of Li ions, as well as their associated diffusion and conductivity properties. Mo et al. employed the electrostatic energy criterion to select the top 10 configurations with the lowest electrostatic energy from a huge number of possible atomic configurations [13]. Then, the first-principles density functional theory (DFT) method was used to determine the thermodynamic ground-state structure (i.e., the most stable atomic configuration). The ab initio molecular dynamics (AIMD) method was then used to study the diffusion of Li ions. The results revealed that Li ions can diffuse in a three-dimensional (3D) network consisting of the c-channel and the ab-plane. In 2012, Xu et al. studied the diffusion of Li ions in one-dimensional (1D) c-channels based on the crystal structure without Li4 sites [14]. They used AIMD simulations to propose a cooperative migration of Li ions in a string-like motion, which was attributed to significant Coulombic repulsion between the ions. In 2013, Ong and Mo et al. expanded the screening space up to the top 30 configurations based on the electrostatic energy criterion [15]. They identified a more stable structure than their previous prediction. The new structure had Li4 sites with P1 space groups and was then incorporated into the Materials Project database (mp-696138). In 2014, Du et al. proposed that a disordered arrangement of tetrahedral GeS₄/P1S₄ could give rise to three types of skeletons, namely, Zig (zigzag in the unit cell), Pa (parallel to the a-axis), and Pc (parallel to the c-axis), as illustrated in Figure 1c–e [16]. The results showed that the Zig-type skeleton, with 2–4 Li ions at the Li4 sites, exhibited the highest stability. In 2016, Bhandari and Bhattacharya employed DFT calculations to re-confirm the existence of Li4 sites [17]. Building upon the most stable Zig skeleton, they investigated the distribution of Li ions in the c-channel and the Li ions’ migration associated with the c-channel, ab-plane, and c-ab. The activation barriers of various possible configurations were evaluated, and then the averaged barriers ($\bar{Ea}$) were found to be consistent with Kamaya et al.’s experimental measurements. In 2018, Oh et al. used first-principles DFT calculations to assess 1000 configurations with low electrostatic energy based on a crystal structure containing Li4 sites [18]. They identified the most thermodynamically stable structure to date, which corresponded to the Zig-type skeleton with a P1 space group. However, the meta-stable Pc-type skeleton facilitated the diffusion of Li ions more effectively, exhibiting a lower diffusion barrier than the Zig-type. In 2022, Dobhal et al. evaluated the thermodynamic stability of the atomic configurations proposed by Mo et al. (P4_2_/nmc and P1) and Oh et al. (P1) using DFT/PBE calculations [19]. The results showed that the structure proposed by Oh et al. was more stable than that proposed by Mo et al., with a total energy difference (ΔE_tot_) of −0.41 eV/cell.

In summary, the identification of stable atomic configurations in LGPS material plays a crucial role in theoretical studies on thermodynamic stability, Li ion diffusion, and other related properties. However, the co-occupancy of Ge/P1 and the defective Li ions’ fractional occupancy result in a huge number of possible atomic configurations, with the sampling space spanning from millions to tens of millions. Currently, the electrostatic energy criterion is the most widely used and efficient approach for accelerating first-principles calculations to determine thermodynamically stable atomic configurations [20,21,22,23]. This criterion enables the rapid calculation of electrostatic energies and the identification of favorable configurations, thereby significantly reducing the computational costs associated with first-principles calculations [24].

In this work, we aimed to obtain thermodynamically stable atomic configurations of LGPS through first-principles calculations and then investigate the effects of various stable configurations, including the skeleton and Li ion distribution, on properties such as Li ion diffusion and conductivity. To achieve this, we utilized the crystal structures reported by Kamaya et al. (without Li4 sites) and Kuhn et al. (with Li4 sites) and employed enumeration algorithms to generate all possible configuration sets with respect to the Pa, Pc, and Zig skeletons. After evaluating the electrostatic energy criterion method, we employed the LAsou method [25], a machine learning- and active-learning-based approach developed by our research group, to explore and predict stable atomic configurations of LGPS. Low-energy stable configurations, predicted using LAsou with diverse skeleton and Li ion distribution, were selected for further property calculations. AIMD simulations were then used to compute the activation energy for Li ion diffusion across a temperature range of 500–900 K. The results were extrapolated to estimate the conductivity at room temperature. In addition, the electronic structure properties of the band gaps were also calculated. Finally, a brief analysis was performed to evaluate the impact of different configurations on Li ion diffusion.

## 2. Methods

### 2.1. Enumerated Configurations and Electrostatic Energies

The crystal structures reported by Kamaya et al. and Kuhn et al. represent two typical experimental results in the study of LGPS. Starting from these experimental structures, we utilized the Supercell [26] package (Supercell v2.1.0) to generate all possible atomic configurations using enumeration algorithms. The cell was set to a 1 × 1 × 1 supercell, containing a total of 50 atoms. To ensure integer numbers of atoms at different sites, we have appropriately adjusted the fractional occupancies of several sites, as specified in the last column of Table S1. For the structure reported by Kamaya et al., the number of combinations for the Li1 sites was $C_{16}^{11}$ = 4368, while that for the Li3 sites was $C_{8}^{5}$ = 56, and that for the co-occupied Ge/P1 sites was $C_{4}^{2}$ = 6. Therefore, the total number of combinations was 4368 × 56 × 6 = 1,467,648, resulting in 91,728 inequivalent (or enumerated) configurations after eliminating duplicates. For the structure reported by Kuhn et al., a total of 2,167,200 enumerated configurations were generated. Clearly, it is not affordable to perform first-principles DFT calculations on all enumerated configurations.

The Supercell package can calculate Ewald-summation-based electrostatic energy [27], which is a Coulomb’s-law-based approach for rapidly estimating the long-range electrostatic energy in ionic crystals and facilitating the determination of their stability. The formal point charges of the four elements in LGPS were adopted as Li (+1), Ge (+4), P (+5), and S (−2). These calculated electrostatic energies were then used to determine the stability orderings of the enumerated configurations. In addition to Supercell, many other packages, such as SOD (SOD v0.52) [28], enumlib (enumlib v2.0.2) [29,30,31], and disorder (disorder v0.6.0) [32], can also be utilized for generating enumerated configurations. Furthermore, the Pymatgen (Pymatgen v2022.7.21) [33] package can conveniently produce atomic configurations and calculate electrostatic energies. However, it should be noted that Pymatgen’s treatment of fractional positions differs from that of Supercell.

### 2.2. Machine Learning- and Active-Learning-Based LAsou Methods

In previous work, we developed a LAsou method based on the machine learning and active learning algorithms, in combination with first-principles calculations. It is an efficient approach for exploring and predicting the thermodynamically stable structures (atomic configurations) of a wide range of solid-state material systems. The acronym LAsou stands for “Large space sampling and Active labeling for searching”, reflecting the core principles of this method. We have successfully tested its efficiency on three distinct chemical-disordered materials, namely, anionic BaSc(O_x_F_1−x_)_3_ (x = 0.667), cationic Ca_1−x_Mn_x_CO_3_ (x = 0.25), and defective ε-FeCx (x = 0.5), with an improvement of 2–3 orders of magnitude when compared to the methods entirely calculated using first-principles DFT [25]. Additionally, we have successfully employed the LAsou method to predict stable atomic configurations of the thermoelectric material Mg_3_Bi_2−x_Sb_x_ (0 < x < 2) [34] and the ternary electride material LaCu_0.67_Si_1.33_ [35] for catalytic applications.

The LAsou method involves the employment of an iterative search process that includes the following steps: (1) Large space configuration sampling: this involves sampling a large configuration space that can be based on either enumerated or randomly generated configurations. (2) Selecting and labelling: from the large configuration space, superior candidates are identified and labelled using criteria such as energies, diversities, and repeatabilities. (3) Energy assessment: the selected candidates will be subjected to first-principles DFT structural relaxation and single-point energy calculations. (4) Machine learning (ML) dataset construction: data points consisting of structure–energy/forces pairs are extracted from the DFT-relaxed trajectories. (5) ML potential training or updating: this involves generating discretized features, selecting an ML model, and using the least-squares algorithm to train the model. For detailed information about the structural features and the ML models utilized in the LAsou method, refer to our previous research [25,34]. (6) ML potential prediction and ordering: the trained ML model is employed to predict the energies of all sampled structures, and then the predicted energy is used as the energetic criterion for selection and labelling. Such an iterative process is repeated from steps (2) to (6) until the convergence condition is satisfied. In the LAsou method, machine learning (ML) is used to construct potential energy models from structural–energy/forces data, providing the stability criterion for rapidly calculating the energies. Active learning (AL) algorithm is employed to continuously improve the reliability and accuracy of these ML potentials. One notable advantage of active learning is its ability to make use of minimal or zero-shot data rather than requiring a large number of training samples to be pre-prepared. It is important for the study of unexplored and novel materials and has garnered considerable attention in the field of material discovery and design [36,37,38,39,40,41,42,43].

In this study, the parameters used in the LAsou method are as follows: the maximum number of generations was 20, the number of selected and labeled samples for each generation was 5, and the number of extracted data points for each DFT-relaxed trajectory was 5. The LBF model was constructed by combining linear ridge regression (LRR) with Gaussian basis function (GBF) features, and then the ensemble-LBF potential was created by combining 5 models using ensemble learning algorithm. The GBF features assume 5 uniform a ∈ [0.1, 2] and 10 uniform b ∈ [0, 5]. The cut-off radius is 6.0 Å, and the percentages of data used in training sets and validation sets are 60% and 40%, respectively.

### 2.3. First-Principles Calculations

The first-principles calculations were performed using density functional theory (DFT) in conjunction with the generalized gradient approximation (GGA) and the Perdew–Burke–Ernzerhof (PBE) [44] functional. The interaction between the ion cores and valence electrons was assessed using the projector-augmented-wave (PAW) [45,46] method, as implemented in the Vienna Ab initio Simulation Package (VASP v5.4.4) [47]. The kinetic energy cut-off was set to 520 eV, and a 4 × 4 × 2 k-point grid was used. The structures were fully relaxed until the residual forces were less than 0.01 eV/Å within the spin-polarized calculation.

In addition, The Heyd–Scuseria–Ernzerhof (HSE) [48] functional was employed to verify the accuracy of the PBE functional associated with Ong et al.’s P4_2_/nmc and P1 configurations [15], as well as Oh et al.’s P1 configuration [18]. Furthermore, the density of states (DOS) and band gap was determined by using the HSE06 [49] functional, where the parameters for mixing and range separation were set to 0.25 and 0.2, respectively.

### 2.4. AIMD Simulations

Aiming to study Li ion diffusion performance, ab initio molecular dynamics (AIMD) simulations were employed. The simulations were carried out using non-spin-polarized calculations with a kinetic energy cut-off of 280 eV and a k-point grid consisting solely of the G-point. The simulations began with an initial temperature of 100 K, followed by a heating process carried out to reach the desired temperature range of 500–900 K via gradually adjusting the velocities over 1000 time steps (2 ps). After that, an equilibrium process was performed for 5000 time steps (10 ps). During the final sampling process, a total duration of 2000 ps was maintained at 500 K, while the diffusion convergence criteria were used between 600 and 900 K, resulting in a duration range of 500 to 1000 ps. All simulations were carried out in the canonical ensemble (NVT) by using Nose–Hoover thermostat [50,51].

## 3. Results and Discussion

### 3.1. Evaluation of the Electrostatic Energy Criterion Method

The main steps of using the electrostatic energy criterion method to obtain the stable configurations are as follows. Firstly, the electrostatic energies are rapidly calculated for all configurations, and the stability orderings are determined based on the magnitudes of those energies. Secondly, a small number of configurations with the lowest electrostatic energies (such as 10, 30, or 1000) are selected for further screening. Finally, first-principles calculations are performed to verify and determine the stable configurations at a high level of accuracy, including both the most stable and meta-stable configurations. In this regard, it is crucial to ensure consistency in the stability orderings between the electrostatic energies and the first-principles energies in order to obtain reliable screening results.

**Figure 2:**
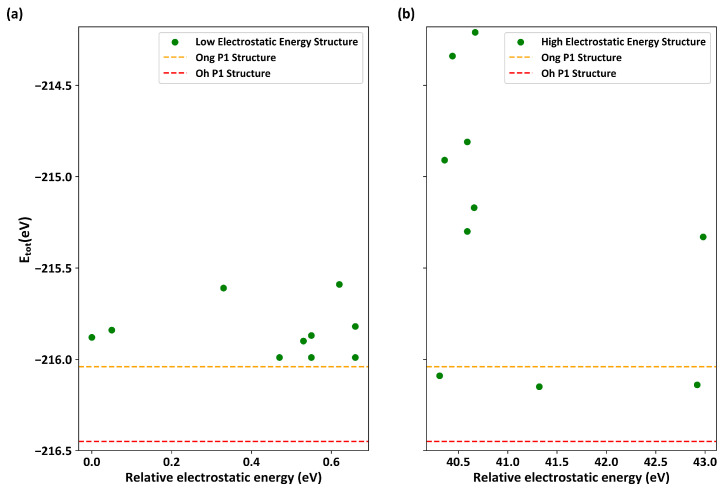
The energy distribution of DFT/PBE relaxed configurations screened using the electrostatic energy criterion based on Kamaya et al.’s experimental crystal structures without Li4 sites. (**a**) The 10 best configurations with the lowest electrostatic energy; (**b**) the 10 worst configurations with the highest electrostatic energy. Here, the horizontal axis represents the relative electrostatic energy (in eV), and the vertical axis represents the total energy of DFT/PBE (in eV). The orange dashed line and red dashed line represent Ong et al.’s and Oh et al.’s reference configurations, respectively.

To evaluate this consistency, we selected the 10 best and worst configurations based on the lowest and highest electrostatic energies out of the 91,728 enumerated configurations generated from Kamaya et al.’s crystal structure. We then performed DFT/PBE structural relaxation calculations. Figure 2 shows the energy distributions resulting from the DFT/PBE relaxation for the 10 configurations with (a) the lowest and (b) the highest electrostatic energies, respectively. In addition, we performed DFT/PBE calculations with the same level of accuracy for Ong et al.’s P1 configuration (represented by the orange dashed line) and Oh et al.’s P1 configuration (represented by the red dashed line) as a reference. In Figure 2a, although the total energy (Etot) is sufficiently low after the DFT calculations, none of the values are superior the results obtained by Ong et al. Figure 2b exhibits a wide range of total energies, in which many configurations display poor stability due to high energies, while some others have lower energy than the results obtained by Ong et al. Moreover, it was observed that none of the 10 lowest electrostatic configurations contain Li4 sites after the DFT calculations were performed. By contrast, Li4 sites were found in the 10 highest configurations. This clearly indicates that the consistency of orderings between simple electrostatic energies and DFT-relaxed energies is not always reliable [23]. Therefore, solely relying on a limited number of low electrostatic configurations for further DFT calculations may lead to the failure to identify or incorrect identification of stable configurations. In addition, we also evaluated the 2,167,200 enumerated configurations generated from Kuhn et al.’s crystal structure, which yielded similar results, as shown in Figure S1.

**Figure 3:**
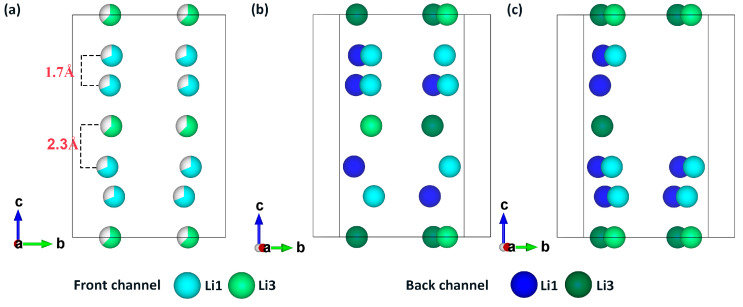
The ionic distribution of Li1 and Li3 sites in the c-channel of LGPS. All other atoms are hidden. (**a**) Schematic representation of the distance between Li1 and Li1 and Li1 and Li3 in the crystal structure; (**b**) (4,4,4,4) distribution of the configuration with the lowest electrostatic energy; and (**c**) (4,6,3,3) distribution of the configuration with the highest electrostatic energy.

Figure 3a shows the distribution of Li1 and Li3 sites within the four c-channels of the crystal structure. The distances between adjacent Li1-Li1 and Li1-Li3 sites are 1.7 Å and 2.3 Å, respectively. After analyzing the 10 best configurations with the lowest electrostatic energy, as shown in Figure 2a, we found that Li ions are uniformly distributed in the c-channels with a (4,4,4,4) configuration, which means that each c-channel in the unit cell contains four Li ions. We employ the notation (k,l,m,n) to simplify the representation of the initial configurations of Li^+^ in the four channels, as shown in Figure 3b. Here, k, l, m, and n denote the number of Li ions in the front-left, back-left, front-right, and back-right channels, respectively. For comparison, we selected one of lowest and one of highest electrostatic configurations, as shown in Figure 3b,c, where the Li ion distributions in the c-channels are (4,4,4,4) and (4,6,3,3), respectively. As is well known, the equilibrium distance between Li-Li atoms typically ranges from 2.2 to 2.4 Å [52]. In the c-channel, if there are too many adjacent Li1-Li1 pairs with a distance of 1.7 Å, the Coulombic repulsion will significantly increase, resulting in a higher electrostatic energy. The former arrangement, (4,4,4,4), is more uniform than the latter, (4,6,3,3), and can minimize the Coulombic repulsion between positively charged Li ions [53]. By contrast, the latter has a denser arrangement, with six Li ions in one of the channels, in which it has more Li1-Li1 pairs and leads to an overall increase in electrostatic energy. Furthermore, such densely distributed configurations undergo significant changes during DFT relaxation. These changes are mainly manifested in lattice distortion (c-axis deformation) or the migration of some Li ions from the c-channel to adjacent new positions in the ab-plane. These notable configuration changes not only facilitate the emergence of new sites but may also contribute to a more stable DFT-relaxed configuration with lower total energy.

When using the electrostatic energy criterion to obtain thermodynamically stable atomic configurations, it is important to be aware of several limitations that may introduce significant errors. Firstly, fixed formal point charges for each element must be manually specified, which might not accurately represent the charge density. Secondly, electrostatic energy only represents the single-point energy under a fixed configuration and does not express the changes in ion positions and/or crystal lattice. Thirdly, electrostatic energy is just one part of the total energy; it is unreasonable to use it as the sole criterion for estimating the total energy and stability.

### 3.2. Stable Atomic Configurations Predicted using the LAsou Method

**Figure 4:**
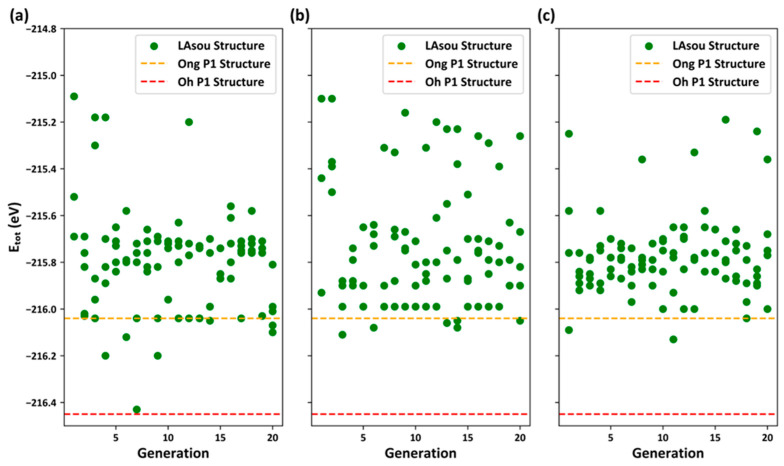
The energy distribution of LAsou results based on Kamaya et al.’s crystal structure for (**a**) the Zig, (**b**) Pa, and (**c**) Pc skeletons. The horizontal axis represents the generations, and the vertical axis represents the total energy of DFT/PBE (in eV).

At this stage, the LAsou method, combined with first-principles DFT calculations, was employed to explore and predict the stable and most stable atomic configurations from the enumerated configurations with the Pa, Pc, and Zig skeletons. Figure 4 illustrates the distribution of DFT/PBE total energy for Kamaya et al.’s enumerated configurations versus the generations. For the Zig skeleton, there was a rapid decrease in total DFT energy in the previous generations, and it reached a stable point of the lowest energy in the seventh generation. No further configurations with lower energy were observed in subsequent searches until the 20th generation. The lowest energy configuration in the seventh generation corresponds to the best result to date achieved by Oh et al., which is a Zig skeleton with Li4 sites and a uniform distribution of Li ions in the c-channel. Nevertheless, due to small discrepancies in the DFT/PBE calculated parameters, there are few differences in the relaxed lattice constants and atomic positions, with a total energy difference (ΔE_tot_) of only 0.01 eV/cell. We have presented the initial un-relaxed and final relaxed crystal structures of the lowest-energy configuration from the seventh generation in Table S2. Hence, one can observe that the LAsou method successfully identified the most stable configuration using only 35 configurations (seven generations × five configurations per generation) in conjunction with DFT calculations. This contrasts with the approximately 1000 configurations exhaustive DFT calculations in Oh et al.’s work [18]. Therefore, a simple comparison reveals that the LAsou method offers a significant speed improvement of nearly 30 times. For the Pa and Pc skeletons, LAsou can successfully predict several superior configurations that are more stable than Ong et al.’s P1 configuration, although none of them are as stable as Oh et al.’s P1 configuration. The findings from various skeletons consistently demonstrate that the Zig skeleton exhibits greater stability compared to the Pa and Pc skeletons, which is in agreement with the previous reports in the literature [16].

As mentioned previously, the 91728 enumerated configurations from Kamaya et al.’s crystal structure do not contain Li4 sites. However, the LAsou method demonstrates high efficiency in predicting the most stable configuration where Li4 sites are occupied by 2 Li ions. Analysis of the initial structure of the optimal configuration reveals a Li ion distribution in the c-channel of (6,4,3,3), with one c-channel containing six Li ions, resulting in significant Coulombic repulsion. After DFT relaxation, the dense Li ions in this channel can migrate through the ab-plane, leading to the formation of Li4 sites. It should be noted that the current initial configuration exhibits remarkably high energy according to the electrostatic energy criterion, making it unsuitable for screening in forward DFT calculations. We have examined the ordering of this initial configuration, which ranked 3023rd among all enumerated configurations and 960th among all Zig configurations. This implies that several hundred configurations must undergo DFT calculations in order to obtain the best result based solely on electrostatic energy ordering. Furthermore, the earliest crystal structure determined by Kamaya et al. can be considered a result of “low resolution”. The LAsou method successfully predicted new Li4 sites that have been experimentally confirmed within a few generations. Thus, the LAsou method provides a novel approach that can assist in the analysis and refinement of experimental crystal structures.

**Figure 5:**
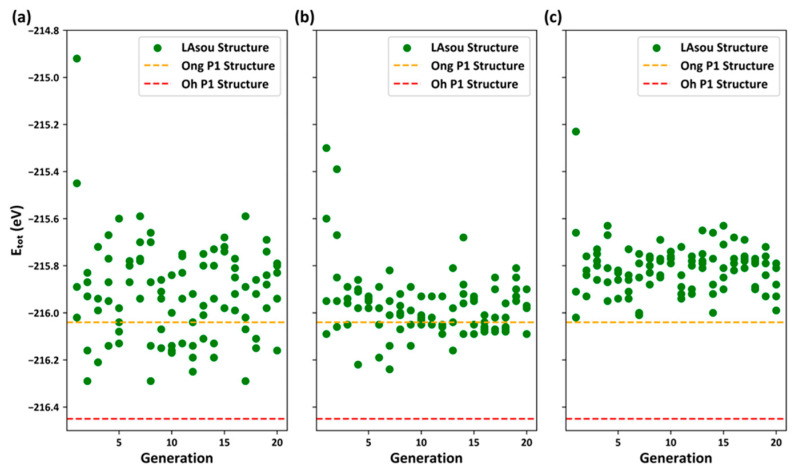
The energy distribution of LAsou results based on Kuhn et al.’s crystal structure for (**a**) the Zig, (**b**) Pa, and (**c**) Pc skeletons. The horizontal axis represents the generations, and the vertical axis represents the total energy of DFT/PBE (in eV).

We further employed the LAsou method to explore the enumerated configurations reported by Kuhn et al. using the same running parameters. Figure 5 illustrates the distribution of the total DFT energy versus generations for the Zig, Pa, and Pc skeletons, respectively. We have also identified several meta-stable configurations within all three skeletons that exhibit greater stability than those reported by Ong et al., although none of them yield a lower total DFT energy than Oh et al. It is noteworthy that the most stable configuration, predicted using the LAsou method based applied to Kamaya et al.’s crystal structure, contains two Li ions at Li4 sites with a fractional occupancy of approximately 0.5, while the Li4 sites in Kuhn et al.’s crystal structure correspond to 0.75, as presented in Table S1. The Supercell package strictly generates enumerated configurations according to 0.75 rather than 0.5. This discrepancy may explain the failure of the LAsou method in predicting the previously obtained best result. To investigate this issue, we adjusted the fractional occupancies of the Li2 and Li4 sites in Kuhn et al.’s crystal structure from 0.75 and 0.75 to 1.0 and 0.5, respectively. Then, the Supercell package was applied to re-generate the enumerated configurations, and the LAsou method was employed to explore and predict the configuration of the Zig skeleton, as demonstrated in Figure S2. As expected, the LAsou method can still successfully predict the best result within just a few generations.

### 3.3. Influence of the Li Ion Distribution on Diffusion and Conductivity

The total number of Li ions in the c-channels of Kamaya et al.’s and Kuhn et al.’s crystal structures are both 20, where the number of Li ions at Li4 sites are 0 and 3, respectively. Interestingly, the most stable configuration predicted by Oh et al. and the LAsou method contains two Li ions at Li4 sites. The number of Li ions at the Li4 sites can represent the disordered distribution of Li ions across different sites, which can significantly impact stability [16,17]. Here, we simply focus on the number of Li ions at the Li4 sites to investigate its influence on Li ion diffusion and conductivity. Based on the prediction of the LAsou method, we selected the most stable configurations (with the lowest DFT energies) with 0, 1, 2, and 3 Li ions at the Li4 sites for the Zig, Pa, and Pc skeletons. These configurations are denoted as Zig_n, Pa_n, and Pc_n, where n represents the number of Li ions (n = 0, 1, 2, 3), resulting in a total of 12 configurations. It should be noted that none of the 20 generations produced a configuration where the Li4 sites are fully occupied by four Li ions. Among the selected configurations, Zig_2 is the most stable configuration predicted using the LAsou method, while the remaining 11 configurations are meta-stable configurations with different skeletons and distributions of Li ions.

After performing AIMD simulations and obtaining the trajectories, the mean square displacement (MSD) was calculated. Then, we could determine the overall diffusion coefficient (D), as well as the anisotropic diffusion coefficients for the c-channel (D^c-channel^) and the ab-plane (D^ab-plane^), through statistical analysis of MSD in different directions. Next, the overall (E_a_) and anisotropic diffusion activation energies (E_a_^c-channel^ and E_a_^ab-plane^) were obtained by fitting the Arrhenius equation from the linear relationship between log D and 1000/T. All the linear fitting results of log D ~ 1000/T for the 12 configurations at different temperatures are presented in Figure S3. Finally, we extrapolated to 300 K to obtain the diffusion activation energy at room temperature and estimated the conductivity at 300 K (σ_RT_) by using the Nernst–Einstein equation. All the aforementioned data analysis and processing steps were performed using the Pymatgen package [33].

Table 1 presents the total DFT energy difference; the overall, c-channel, and ab-plane diffusion activation energies; and the estimated conductivity at 300 K. These results clearly demonstrate that the skeletons and Li ion distributions in LGPS have a significant impact on thermodynamic stability, diffusion activation energy, and conductivity. Among them, the stability ordering of the skeleton and the ionic diffusivity ordering of Li ions are both in agreement with previously reported results [16,18]. Meanwhile, Li ion diffusion is more favorable in the c-channel compared to the ab-plane, which can be attributed to the enhanced 1D migration of dense Li ions throughout the entire c-channel and the corresponding cooperative effects [14].

Our predicted Zig_2 configuration is consistent with the P1 structure reported by Oh et al., exhibiting comparable diffusion activation energy and conductivity [18,19]. In addition, the predicted Zig_1 configuration shares similarities with the P1 structure reported by Ong et al. in terms of stability, diffusion activation energy, and conductivity [15]. However, owing to differences in the running parameters in the AIMD simulations, there may be slight discrepancies in the conductivity values [54]. In practice, overall performance is determined statistically by considering a range of stable and meta-stable configurations in order to reproduce experimental results [17]. To assess the significance of these stable configurations and obtain more-reliable statistical diffusion properties, we combined all 12 configurations to calculate the averaged activation energy using the following equation:

(1) $$\left\langle E_{a}\rangle =\sum_{i=1}^{N}\;(p_{i}E_{a,i}\right)$$

where $E_{a,i}$ is the activation energy of the i-th configuration, and $p_{i}$ is the stability-dependent probability of this configuration [28]

(2) $$p_{i}=\frac{1}{Z}\exp(-\frac{\Delta E_{i}}{k_{B}T})$$

The normalization factor Z is defined as

(3) $$Z=\sum_{i=1}^{N}\exp(-\frac{\Delta E_{i}}{k_{B}T})$$

where $\Delta E_{i}$ denotes the DFT energy difference compared to the most stable Zig_2 configuration.

**Figure 6:**
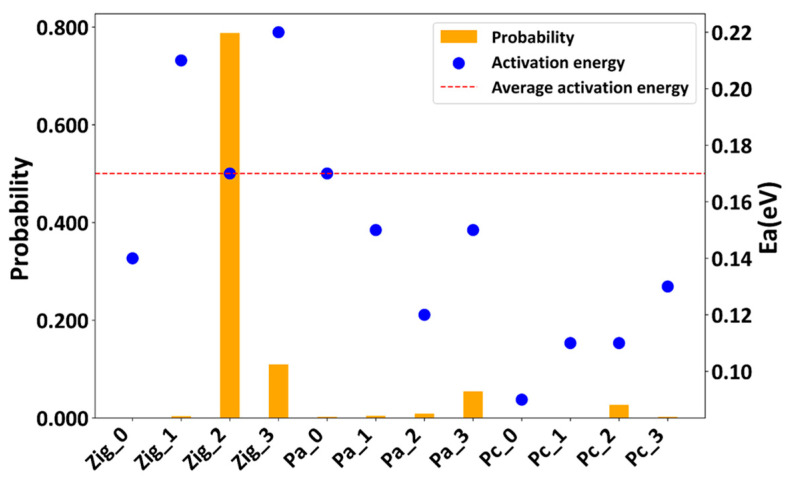
The stability-dependent probabilities and activation energies of the 12 stable configurations. The red dashed line represents the averaged activation energy at 400 K.

Kato et al. have shown that the experimentally measured diffusion activation energy within the temperature range of 322–673 K was approximately 0.17 eV [12]. We selected 400 K as a reference to calculate the stability-dependent probability and the averaged diffusion activation energy, as shown in Figure 6. The most stable Zig_2 configuration exhibits a high probability of 0.978, while the cumulative probability of all other configurations is 0.022. The weighted diffusion activation energy from the probabilities of all 12 configurations is 0.17 eV, which closely matches the experimentally reported value.

Additionally, we performed density-of-states (DOS) calculations for all 12 configurations using the DFT/PBE and DFT/HSE06 methods. The results are illustrated in Figures S4 and S5. The band gap obtained from the DOS calculations can directly correlate with the electrochemical window of solid-state electrolytes [55,56]. The band gap range obtained from DFT/PBE is 2.29–2.70 eV, while DFT/HSE06 yields a range of 3.39–3.83 eV. These variations among configurations can give rise to a band gap difference of up to 0.4–0.5 eV, where it highlights the significant influence of different configurations on the electronic structure.

## 4. Conclusions

In this study, we employed the LAsou method, in combination with first-principles calculations, to determine the thermodynamically stable atomic configurations of the LGPS-type solid-state electrolyte. Subsequently, AIMD simulations were performed to investigate Li ion diffusion performance based on 12 stable and meta-stable configurations. The main findings and conclusions of this work are as follows:

(1) Using the experimental crystal structures provided by Kamaya et al. and Kuhn et al., we utilized the Supercell package to generate 91,728 and 2,167,200 enumerated configurations, respectively. These configurations were categorized into Zig, Pa, and Pc skeletons based on the disordered arrangements of GeS_4_ and P1S_4_ tetrahedra. The presence of co-occupancy, defects, doping, and other fractional occupancies in crystal structures can result in an exceptionally large number of possible configurations in sampling (combinatorial) space, posing a big challenge for both experimental and theoretical research.
(2) The electrostatic energy criterion, although very useful in screening a small subspace within a huge sampling space, has notable limitations. For example, configurations with low electrostatic energy often adhere to the lower Coulombic repulsion rule and tend to have a uniform distribution. Additionally, the small size of the subspace may result in the failure to obtain superior results in electrostatic regions with high energy.
(3) In contrast to the electrostatic energy criterion, the LAsou method utilizes data-driven machine learning and active-learning algorithms. These algorithms facilitate iterative sampling and labeling in a large space, ultimately identifying stable configuration with minimal computational cost. For the Zig skeletons, only 35 configurations calculated using DFT were necessary to achieve similar theoretical results to those of Oh et al., who performed exhaustive DFT calculations on nearly 1000 configurations. Furthermore, the LAsou method successfully reproduced the new Li4 sites based on the low-resolution crystal structure reported by Kamaya et al. This suggests that the LAsou method can also contribute to the refinement of experimental crystal structures.
(4) Based on the predicted stable configurations of Zig_n, Pa_n, and Pc_n (n = 0, 1, 2, 3), we performed AIMD simulations to investigate Li ion diffusion at temperatures ranging from 500 to 900 K. The results highlighted the significant impact of the skeleton and Li ion distribution on diffusion activation energy, ionic conductivity, and electronic structure properties. Although various stable configurations may co-exist, the overall weighted average is closer to the experimental results, where the most stable configuration makes the greatest contributions.

In addition to the electrostatic energy criterion, the machine leaning- and active-learning-based LAsou method, in combination with first-principles calculations, offers a novel and efficient approach for predicting thermodynamically stable atomic configurations. Furthermore, the LAsou method proves beneficial for experimental crystal refinement and theoretical structure modeling and holds promise for facilitating the design of LGPS-type and new solid-state electrolyte materials in the future.

## Figures and Tables

**Table 1:** Total DFT energy differences (ΔEtot, in eV) and diffusion activation energies (Ea, in eV) overall, for the c-channel, and for the ab-plane, and the estimated conductivity at 300K (σ, in mS/cm) of the 12 stable configurations.

Configuration	ΔE_tot_	$E_{a}^{\mathrm{overall}}$	$E_{a}^{\mathrm{c-channel}}$	$E_{a}^{\mathrm{ab-plane}}$	σ
Zig_0	0.62	0.14 ± 0.023	0.12 ± 0.037	0.22 ± 0.022	95
Zig_1	0.38	0.21 ± 0.028	0.19 ± 0.034	0.27 ± 0.015	23
Zig_2	0	0.17 ± 0.008	0.15 ± 0.008	0.25 ± 0.026	43
Zig_3	0.14	0.22 ± 0.019	0.20 ± 0.020	0.27 ± 0.005	15
Pa_0	0.42	0.17 ± 0.009	0.15 ± 0.014	0.24 ± 0.011	45
Pa_1	0.37	0.15 ± 0.027	0.13 ± 0.035	0.22 ± 0.015	80
Pa_2	0.32	0.12 ± 0.019	0.09 ± 0.025	0.18 ± 0.035	172
Pa_3	0.19	0.15 ± 0.023	0.12 ± 0.031	0.22 ± 0.022	84
Pc_0	0.54	0.09 ± 0.016	0.07 ± 0.023	0.15 ± 0.013	332
Pc_1	0.39	0.11 ± 0.031	0.09 ± 0.036	0.17 ± 0.023	243
Pc_2	0.24	0.11 ± 0.014	0.08 ± 0.016	0.16 ± 0.014	207
Pc_3	0.42	0.13 ± 0.017	0.11 ± 0.019	0.16 ± 0.02	148

## Data Availability

Data are contained within the article.

## Supplementary Materials

The following supporting information can be downloaded at https://www.mdpi.com/article/10.3390/ma17081810/s1. Table S1. Experimental crystal structures of Kamaya et al. and Kuhn et al. The last column is the adjusted occupation used in this article. Table S2. The most stable configuration predicted by the LAsou method. Figure S1. The energy distribution of DFT/PBE relaxed configurations screened by the electrostatic energy criterion based on Kuhn et al.’s experimental crystal structures with Li4 sites. Figure S2. The energy distribution of LAsou results for the Zig skeleton of Kuhn et al.’s crystal structure, where the fractional occupancies of Li2 and Li4 sites are adjusted from 0.75 and 0.75 to 1.0 and 0.5, respectively. Figure S3. Arrhenius plots of the diffusion coefficients (log D) as a function of temperature (1000/T) for 12 stable configurations from the AIMD simulations. Figure S4. The partial density of states (PDOS) for 12 stable configurations under DFT/HSE06 functional. The valence band maximum is set at 0 eV. Figure S5. The partial density of states (PDOS) for 12 stable configurations under DFT/PBE functional. The valence band maximum is set at 0 eV. References [5,7] are cited in the supplementary materials.
