# Supplementary material for: Machine Learning-Accelerated First-Principles Study of Atomic Configuration and Ionic Diffusion in Li10GeP2S12 Solid Electrolyte
Source: Materials (Basel). 2024 Apr 15;17(8):1810. doi: 10.3390/ma17081810 (PMC11051406; doi:10.3390/ma17081810)
Supplement: Supplementary file 1 [file materials-17-01810-s001.zip › materials-2943916-supplementary.pdf]

Supporting Information

Machine Learning-Accelerated First-Principles Study of Atomic Configuration and Ionic Diffusion in $Li_{10}GeP_2S_{12}$ Solid Electrolyte

Changlin Qi[1,2] ,Yuwei Zhou[1,3*], Xiaoze Yuan[2,4], Qing Peng[4,5,6*], Yong Yang[1,3], Yongwang Li[3], Xiao-Dong Wen[1,2,3]

1. State Key Laboratory of Coal Conversion, Institute of Coal Chemistry, Chinese Academy of Sciences, Taiyuan, 030001, China
2. University of Chinese Academy of Sciences, No. 19A Yuquan Road, Beijing, 100049, P. R. China
3. National Energy Center for Coal to Clean Fuels, Synfuels China Co., Ltd., Huairou District, Beijing 101400, P. R. China
4. State Key Laboratory of Nonlinear Mechanics, Institute of Mechanics, Chinese Academy of Sciences, Beijing 100190, China
5. Center of Materials Science and Optoelectronics Engineering, University of Chinese Academy of Sciences, Beijing, 100049, China
6. Guangdong Aerospace Research Academy, Guangzhou 511458, China

*Corresponding authors
E-mail addresses: zhouyuwei_kt@163.com; pengqing@imech.ac.cn;

Table S1 Experimental crystal structures of Kamaya et al. and Kuhn et al. The last column is the adjusted occupation used in this article.

Atom site	Wyckoff position	x	y	z	Occ.	Adjusted Occ.
Kamaya et al.'s crystal structure (P4$_2$/nmc, *a* = *b* = 8.69407Å, *c* = 12.5994Å)[1]						
Li1	16h	0.2563(5)	0.2718(3)	0.1832(3)	0.691(5)	0.6875
Li2	4d	0	1/2	0.9446(2)	1.0	1.0
Li3	8f	0.2463(5)	=x(Li3)	0	0.643(5)	0.625
Li4	——	——	——	——	——	——
Ge	4d	0	1/2	0.6907(6)	0.515(5)	0.5
P1	4d	0	1/2	0.6907(6)	0.485(5)	0.5
P2	2b	0	0	1/2	1.0	1.0
S1	8g	0	0.184(2)	0.410(2)	1.0	1.0
S2	8g	0	0.299(2)	0.095(2)	1.0	1.0
S3	8g	0	0.699(2)	0.791(2)	1.0	1.0
Kuhn et al.'s crystal structure (room temperature, P4$_2$/nmc, *a* = *b* = 8.7187Å, *c* = 12.6385Å)[2]						
Li1	16h	0.267(3)	0.273(3)	0.194(2)	0.466(18)	0.5
Li2	4d	0	1/2	0.9454(12)	0.86(6)	0.75
Li3	8f	0.248(2)	=x(Li3)	0	0.74(5)	0.75
Li4	4c	0	0	0.251(2)	0.81(7)	0.75
Ge	4d	0	1/2	0.69154(7)	0.5	0.5
P1	4d	0	1/2	0.69154(7)	0.5	0.5
P2	2b	0	0	1/2	1.0	1.0
S1	8g	0	0.18857(13)	0.40707(10)	1.0	1.0
S2	8g	0	0.29557(14)	0.09727(9)	1.0	1.0
S3	8g	0	0.69901(13)	0.79168(9)	1.0	1.0

Table S2 The most stable configuration predicted by the LAsou method

Initial un-relaxed configuration (generated by Supercell package)

Lattice Parameters					
a (Å)	b (Å)	c (Å)	α (°)	β (°)	γ (°)
8.690	8.690	12.599	90.00	90.00	90.00

Atomic positions							
Element	a	b	c	Element	a	b	c
Li	0.256	0.272	0.183	S	0.000	0.184	0.410
Li	0.744	0.728	0.183	S	0.316	0.500	0.910
Li	0.728	0.744	0.817	S	0.000	0.816	0.410
Li	0.272	0.256	0.817	S	0.684	0.500	0.910
Li	0.272	0.744	0.817	S	0.500	0.316	0.090
Li	0.756	0.772	0.317	S	0.816	0.000	0.590
Li	0.728	0.256	0.817	S	0.500	0.684	0.090
Li	0.744	0.272	0.183	S	0.184	0.000	0.590
Li	0.772	0.756	0.683	S	0.000	0.299	0.095
Li	0.256	0.728	0.183	S	0.201	0.500	0.595
Li	0.228	0.244	0.683	S	0.000	0.701	0.095
Li	0.000	0.500	0.945	S	0.799	0.500	0.595
Li	0.000	0.500	0.445	S	0.500	0.201	0.405
Li	0.500	0.000	0.555	S	0.701	0.000	0.905
Li	0.500	0.000	0.055	S	0.500	0.799	0.405
Li	0.254	0.746	0.500	S	0.299	0.000	0.905
Li	0.754	0.754	0.000	S	0.000	0.699	0.791
Li	0.746	0.254	0.500	S	0.801	0.500	0.291
Li	0.254	0.254	0.500	S	0.000	0.301	0.791
Li	0.746	0.746	0.500	S	0.199	0.500	0.291
Ge	0.000	0.500	0.691	S	0.500	0.801	0.709
Ge	0.500	0.000	0.309	S	0.301	0.000	0.209
P	0.000	0.500	0.191	S	0.500	0.199	0.709
P	0.500	0.000	0.809	S	0.699	0.000	0.209
P	0.000	0.000	0.500				
P	0.500	0.500	0.000				

Final relaxed configuration (calculated by VASP 5.4.4 package)

Lattice Parameters					
a (Å)	b (Å)	c (Å)	α (°)	β (°)	γ (°)
8.655	8.811	13.077	90.07	89.63	89.52

Atomic positions							
Element	a	b	c	Element	a	b	c
Li	0.252	0.271	0.166	S	-0.026	0.177	0.411
Li	0.666	0.716	0.254	S	0.305	0.505	0.914
Li	0.709	0.656	0.753	S	-0.017	0.800	0.407
Li	0.278	0.247	0.867	S	0.697	0.506	0.912
Li	0.267	0.733	0.819	S	0.499	0.300	0.084
Li	0.980	0.987	0.266	S	0.793	-0.013	0.595
Li	0.737	0.254	0.830	S	0.510	0.687	0.095
Li	0.731	0.263	0.183	S	0.176	-0.032	0.594
Li	0.970	0.962	0.738	S	-0.012	0.309	0.099
Li	0.258	0.731	0.177	S	0.201	0.480	0.570
Li	0.232	0.229	0.651	S	-0.010	0.692	0.109
Li	0.010	0.500	-0.048	S	0.777	0.496	0.594
Li	-0.028	0.504	0.448	S	0.485	0.198	0.426
Li	0.481	-0.013	0.552	S	0.703	-0.015	0.893
Li	0.485	-0.005	0.064	S	0.495	0.782	0.408
Li	0.241	0.732	0.488	S	0.311	-0.024	0.909
Li	0.747	0.745	0.002	S	-0.006	0.683	0.791
Li	0.732	0.243	0.512	S	0.802	0.481	0.292
Li	0.229	0.297	0.417	S	0.018	0.275	0.780
Li	0.735	0.745	0.505	S	0.191	0.496	0.278
Ge	0.001	0.486	0.681	S	0.485	0.809	0.710
Ge	0.487	0.002	0.318	S	0.279	0.004	0.214
P	-0.011	0.495	0.194	S	0.491	0.190	0.734
P	0.499	-0.013	0.812	S	0.691	-0.008	0.209
P	-0.015	-0.013	0.502				
P	0.499	0.496	0.002				

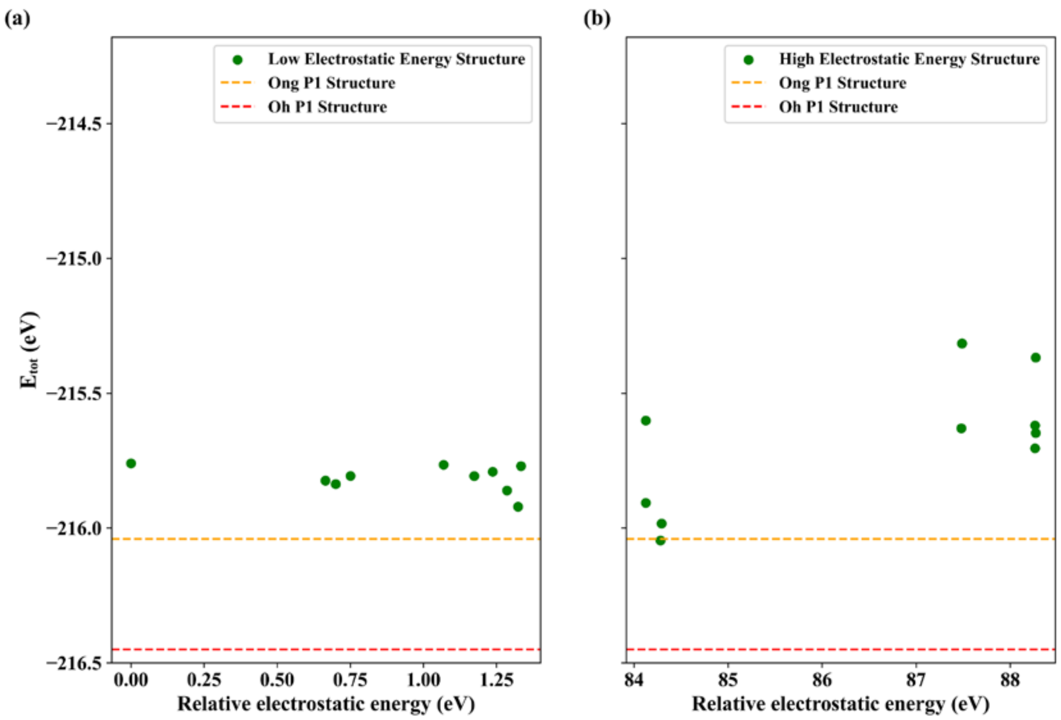

Figure. S1 The energy distribution of DFT/PBE relaxed configurations screened by the electrostatic energy criterion based on Kuhn et al.'s experimental crystal structures with Li4 sites. (a) The best 10 configurations with the lowest electrostatic energy, (b) the worst 10 configurations with the highest electrostatic energy. Where the horizontal axis represents the relative electrostatic energy (in eV), and the vertical axis represents the total energy of DFT/PBE (in eV). The orange dashed line and red dashed line represent the Ong et al.'s and Oh et al.'s reference configuration, respectively.

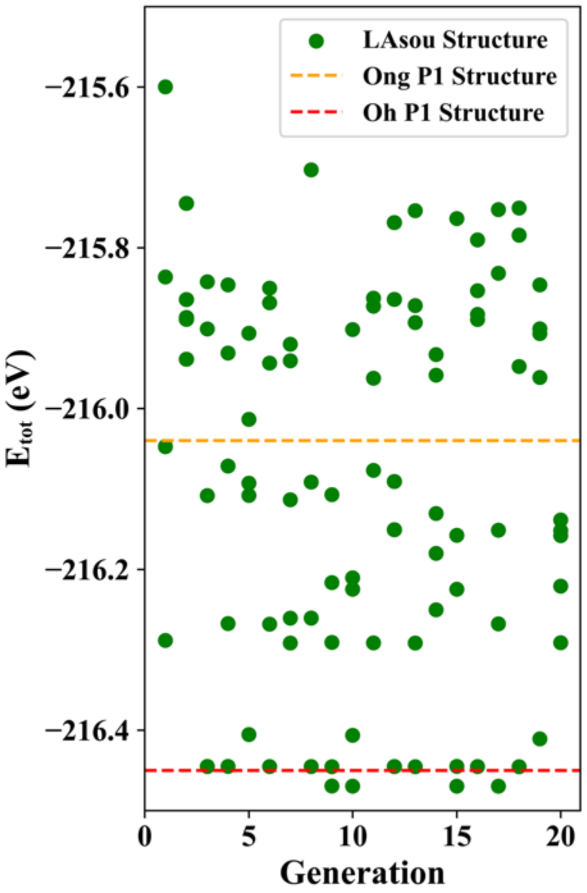

Figure S2 The energy distribution of LAsou results for the Zig skeleton of Kuhn et al.'s crystal structure, where the fractional occupancies of Li2 and Li4 sites are adjusted from 0.75 and 0.75 to 1.0 and 0.5, respectively.

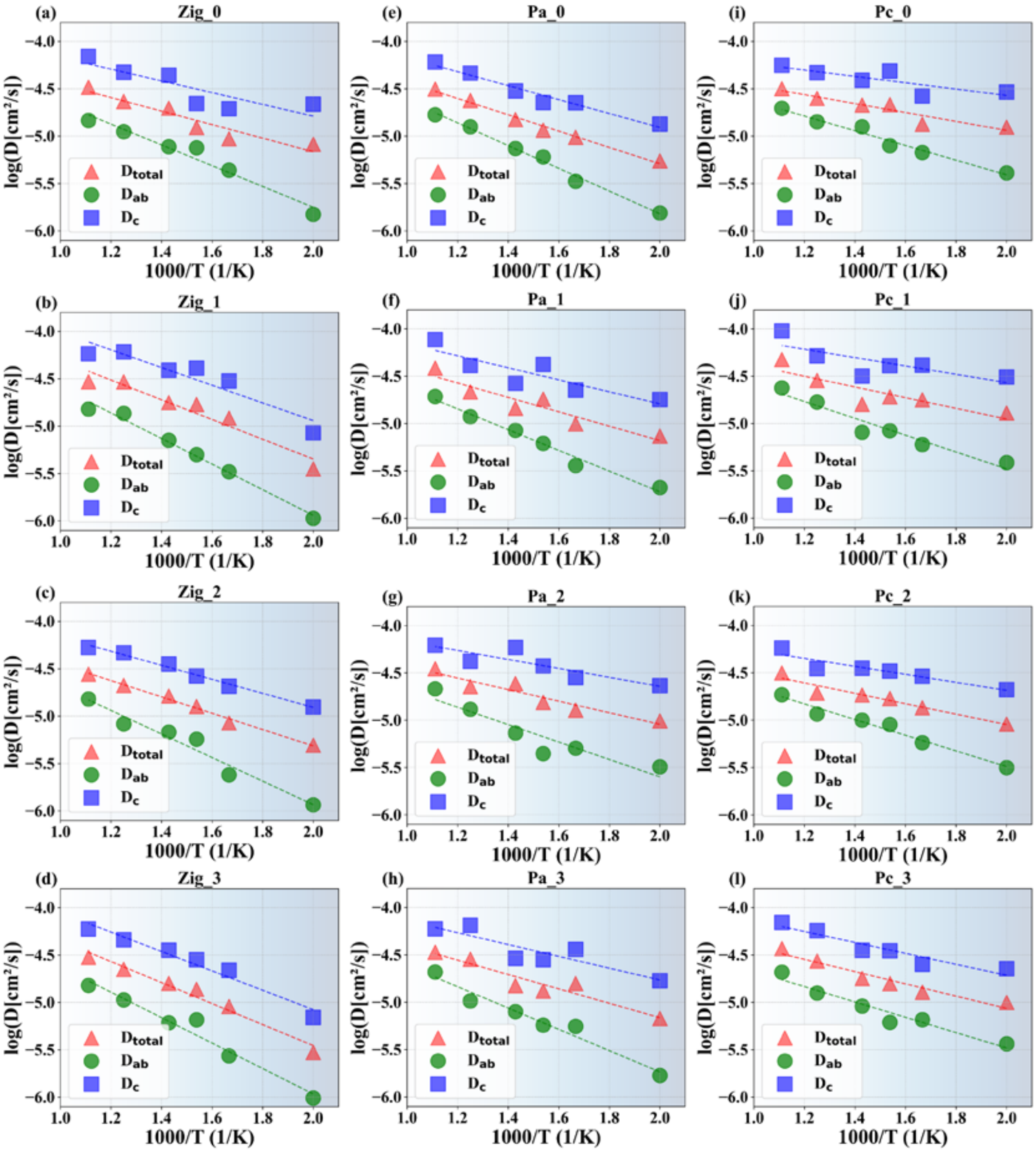

Figure S3 Arrhenius plots of the diffusion coefficients (log D) as a function of temperature (1000/T) for 12 stable configurations from the AIMD simulations.

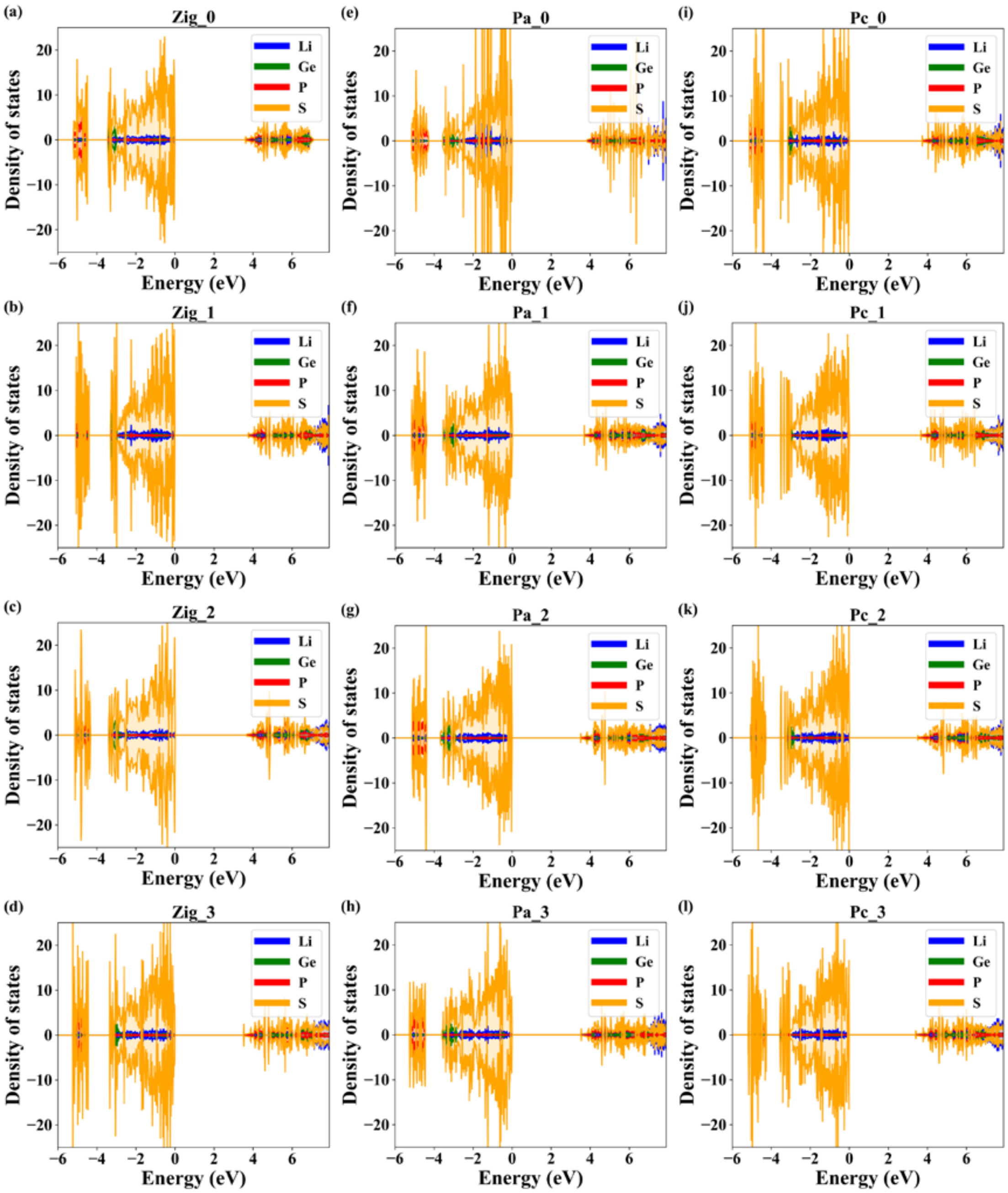

Figure S4 The partial density of states (PDOS) for 12 stable configurations under DFT/HSE06 functional. The valence band maximum is set at 0 eV.

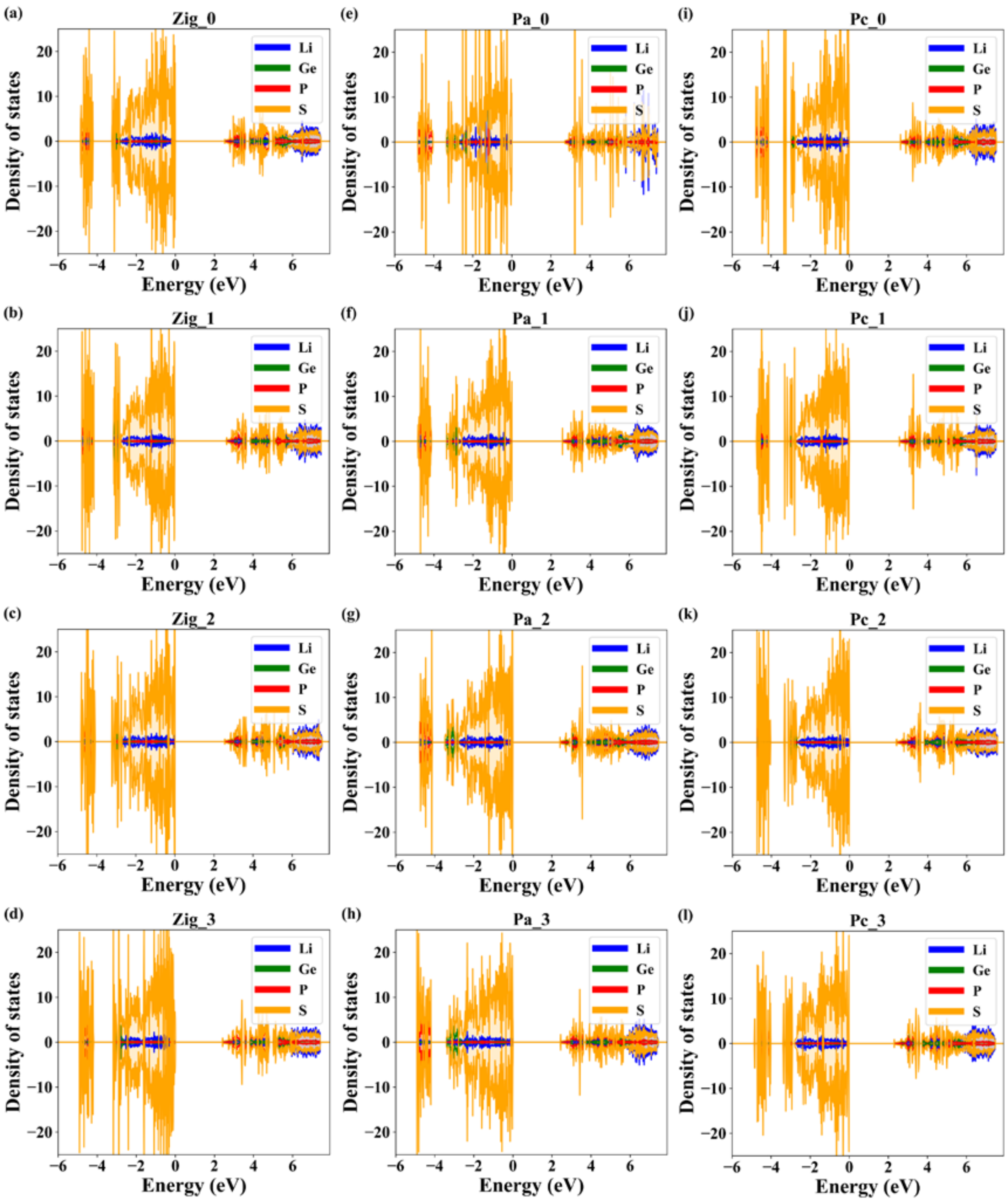

Figure S5 The partial density of states (PDOS) for 12 stable configurations under DFT/PBE functional. The valence band maximum is set at 0 eV.

References:

1. N. Kamaya, K. Homma, Y. Yamakawa, M. Hirayama, R. Kanno, M. Yonemura, T. Kamiyama, Y. Kato, S. Hama, K. Kawamoto and A. Mitsui, *Nat Mater*, 2011, **10**, 682-686.
2. A. Kuhn, J. Kohler and B. V. Lotsch, *Phys Chem Chem Phys*, 2013, **15**, 11620-11622.
